# Therapeutic Power of Oxygen Gas

**Published:** 1871-02

**Authors:** T. D. Crothers


### ARTICLE VI.

### *Therapeutic Power of Oxygen Gas.*

By T. D. Crothers. M. D.

Oxygen has been used as a remedy in disease over a century. The difficulty of separating it from the air, and using it at the bed-side of a patient with its cost, have been obstacles preventing its introduction into general practice. Now, by the process of "Lessis du Motay," immense quantities can be procured, and sent to all parts of the country at trifling cost, in compressed cylinders. The phenomena of life is kept up by nutrition, and absorption of oxygen gas from the air. Oxygen sustains the most intimate relation to life. All other elements may be withdrawn and life will continue for a time; but, if oxygen is withheld, death follows. The secretion and excretion of every atom in the body depends upon the pressure of oxygen. The chemical action of oxygen, on the elements of food, is the ultimate cause of all vitality. Oxygen, and all the elements of food, are taken into the body through the channels of the blood. This fluid not only carries oxygen to the ultimate parts of the body, but is renewed by it, and depends upon it for force and power. When we give iron it is to increase the absorbing power of the blood for oxygen. The true tonic is oxygen. When iron is given fresh air must be increased, or the remedy will fail. A condition of health depends more on the amount of oxygen absorbed than upon nutrition. The absorbing power of the blood may be impaired. Here Dr. Smith, of New York, suggests that, "a deficient absorbing power may be supplimented by an increased supply of the material absorbed." And this explains some of the remarkable results from oxygen especially phthisis. Where the disease is both of the respiration and nutrition of the body, here oxygen not only aids the blood in bringing material to be built up, but supples the building up power, and lessens the increased action of the lungs to supply this want from the atmosphere. Experience does not confirm the theory that oxygen gas, in contact with inflamed and ulcerated surfaces, will increase inflammatory action. Dr. A. H. Smith, of New York, the highest authority on this subject, has recently given 1,100 gallons of pure oxygen gas in 48 hours with no ill effects. The pulse, after inhaling oxygen, becomes steady and regular, often increased in frequency a few beats. The temperature decreases or remains the same. Oxygen is applicable, says Dr. Smith, to two classes of diseases—one in which respiration is at fault, and the other in which both respiration and nutrition are defective.

Under the first class are included Asthma, Emphysema, Croup, Diphtheria, Capillary Bronchitis, Pneumonia, Poisoning by Opium. Astonishing cures have followed its administration in each of these diseases. In asthma the paroxysms will be relieved, and a cure will follow in a very large per cent. of all cases. In capillary bronchitis and emphysema, its effects may be depended upon. In pneumonia of a typhoid type, the results are very gratifying, if carefully used by judicious men. In a low grade of fever, with anæmia, no remedy will act so promptly. In one case of my own, convalescence was established on the fourth day after the administration began. In a severe case of asthma, which had resisted all medication for years, complete relief followed after two inhalations of 6 gallons each. In dispnœa it is almost a specific; and if of no value in any other disease, its value here would establish it as indispensible.

In the second class of diseases, in which both respiration and nutrition are defective, phthisis stands first. In this disease oxygen is the most valuable remedy we possess. It has been used more in this disease than any other, with results exceeding all expectation. One case under my care, the patient gained fourteen pounds in fifty days, with a rapid convalescence, which are strong indications of a complete cure.—*Buffalo Medical Journal.*

